# Supplementary figures and images for: Identification and Characterization of Multiple Paneth Cell Types in the Mouse Small Intestine
Source: Cells. 2024 Aug 27;13(17):1435. doi: 10.3390/cells13171435 (PMC11394207; doi:10.3390/cells13171435)

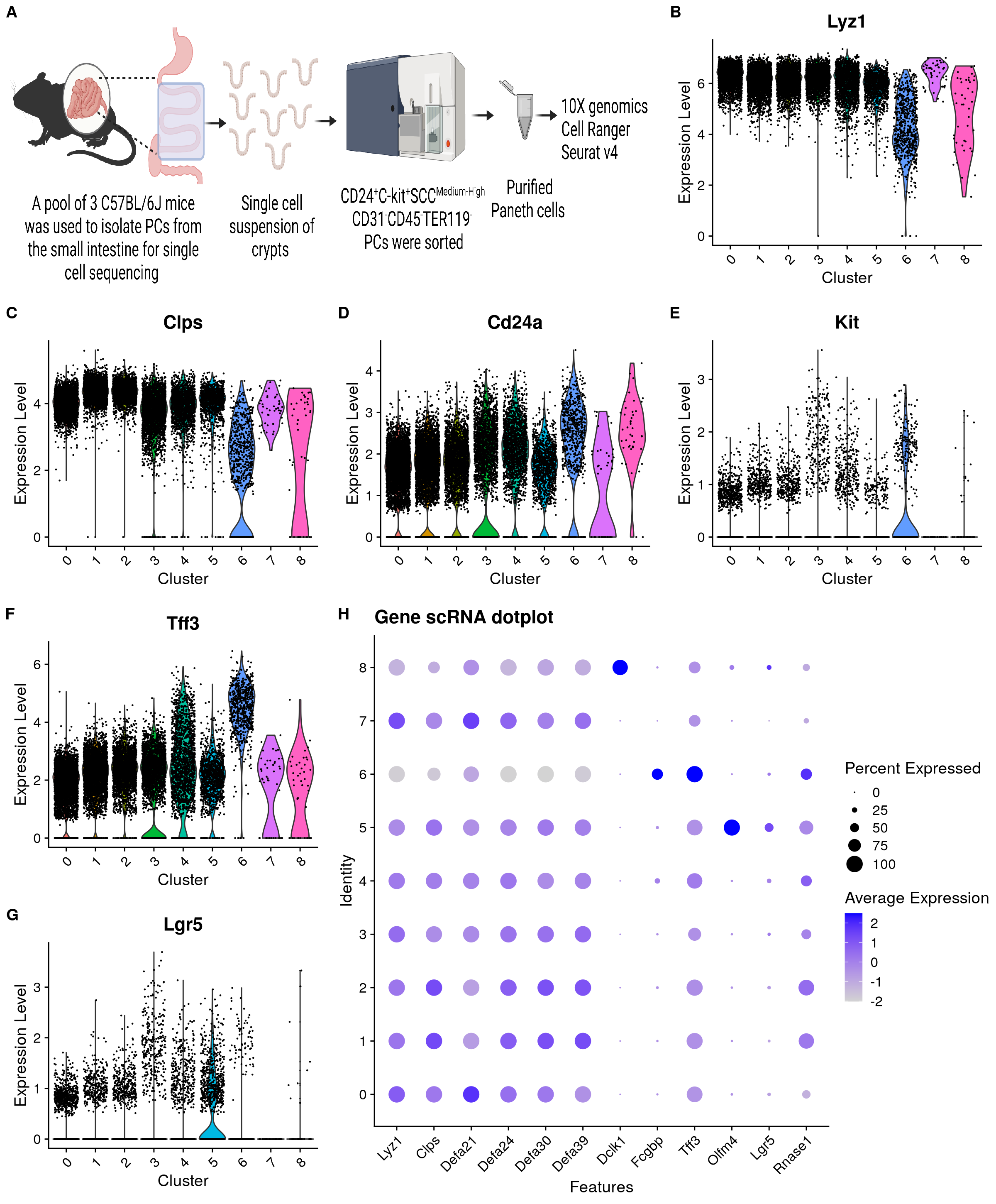

Supplement: Supplementary file 1 [file cells-13-01435-s001.zip › SupFigure_1.png]

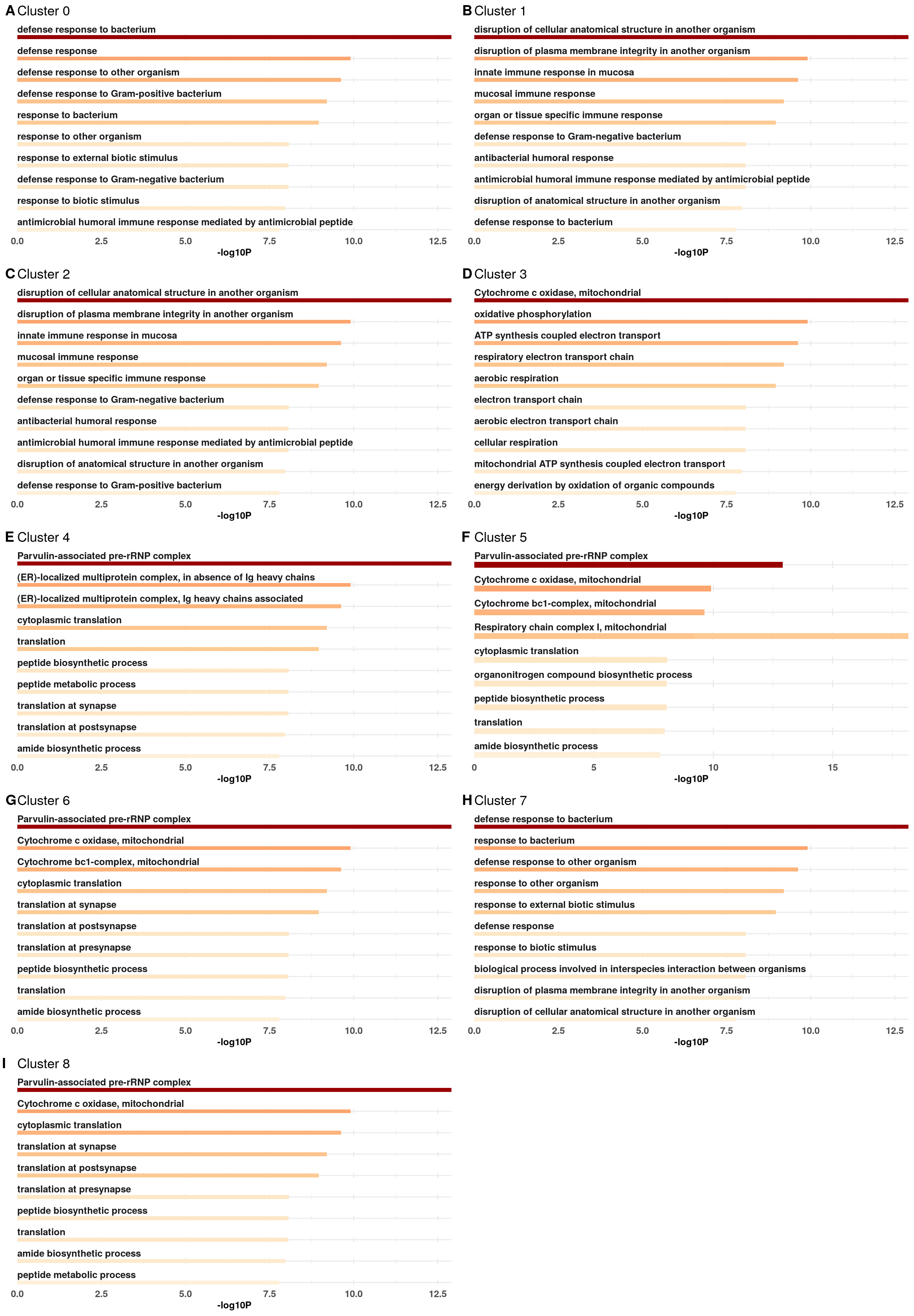

Supplement: Supplementary file 1 [file cells-13-01435-s001.zip › SupFigure_2.png]

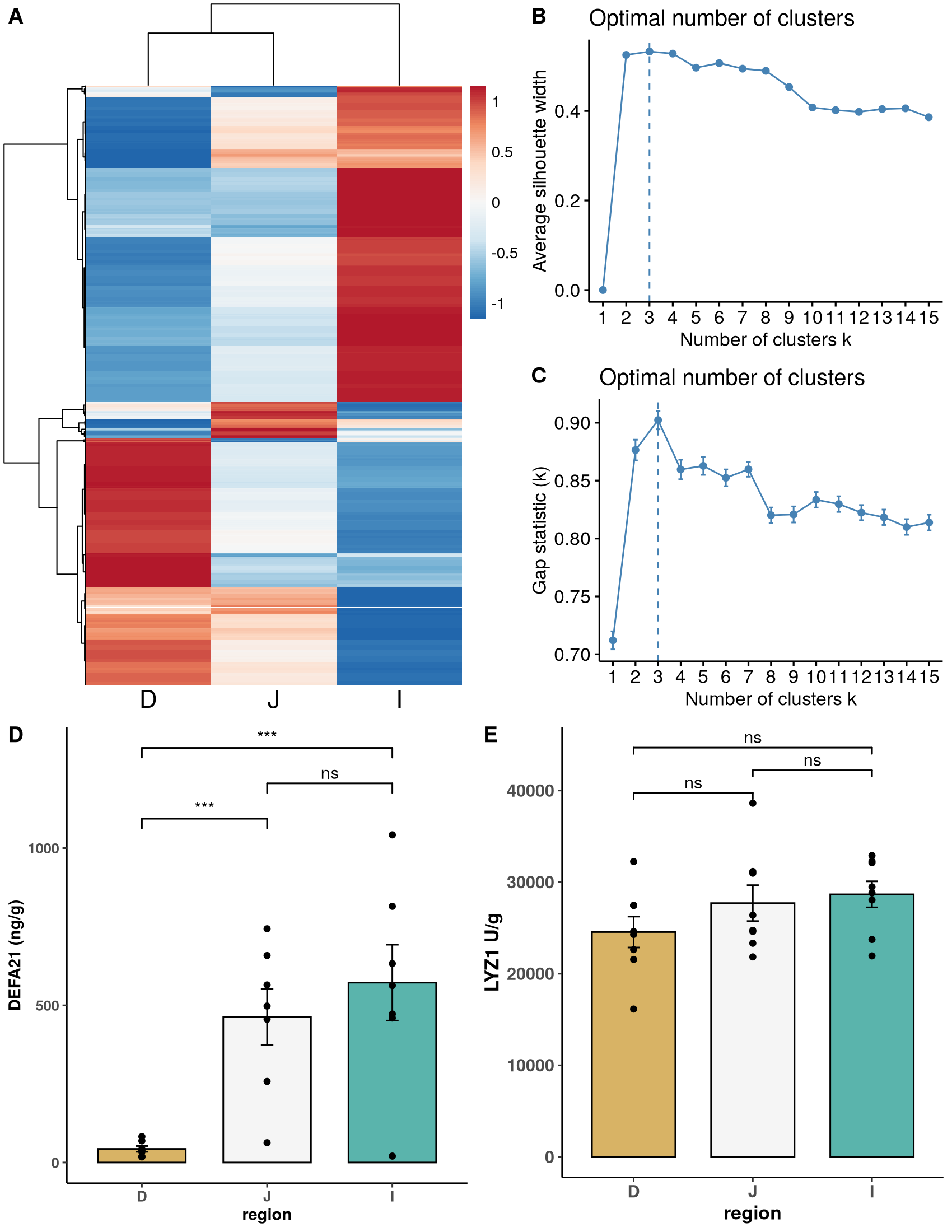

Supplement: Supplementary file 1 [file cells-13-01435-s001.zip › SupFigure_3.png]

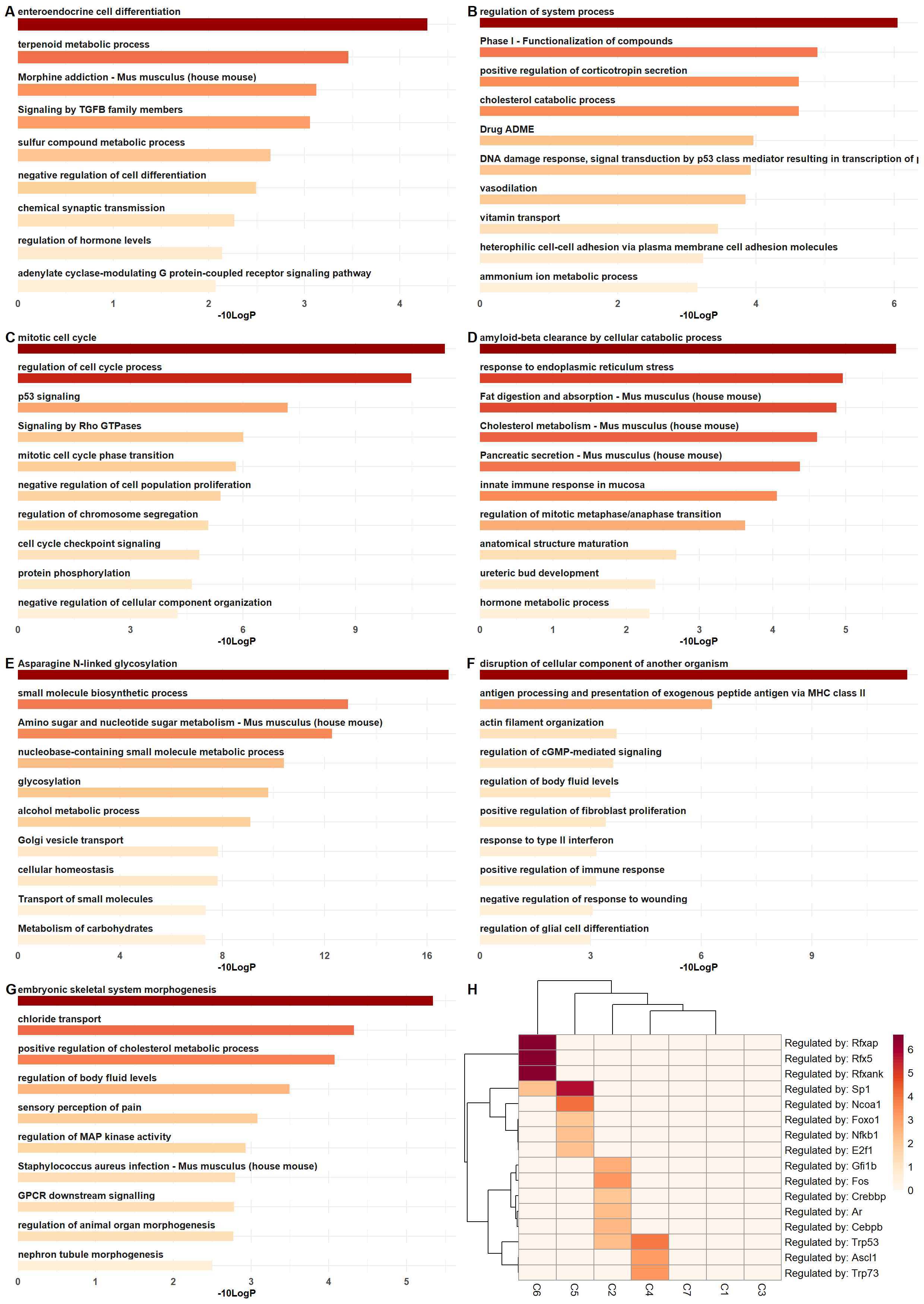

Supplement: Supplementary file 1 [file cells-13-01435-s001.zip › SupFigure_4.png]

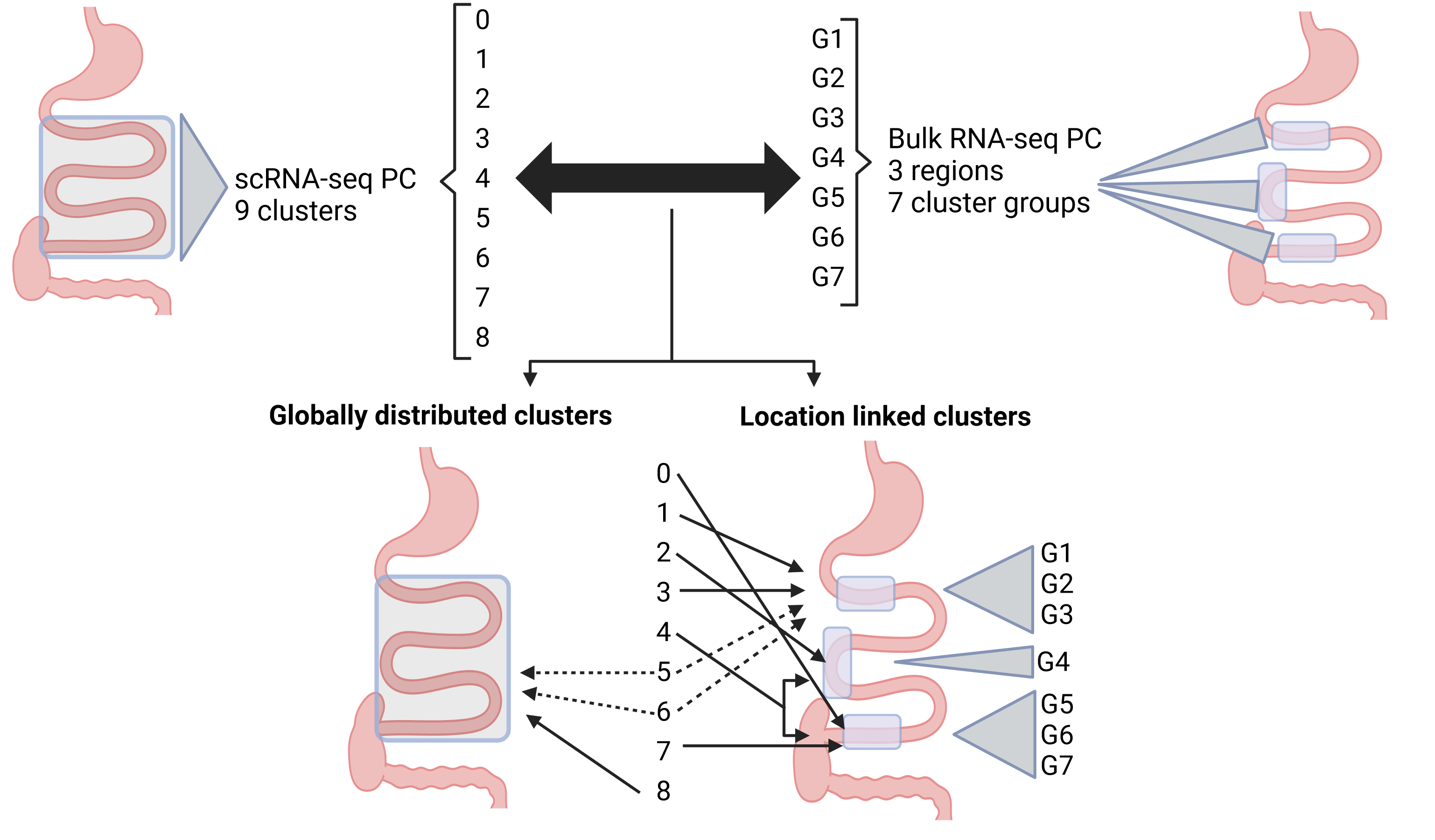

Supplement: Supplementary file 1 [file cells-13-01435-s001.zip › SupFigure_5.png]

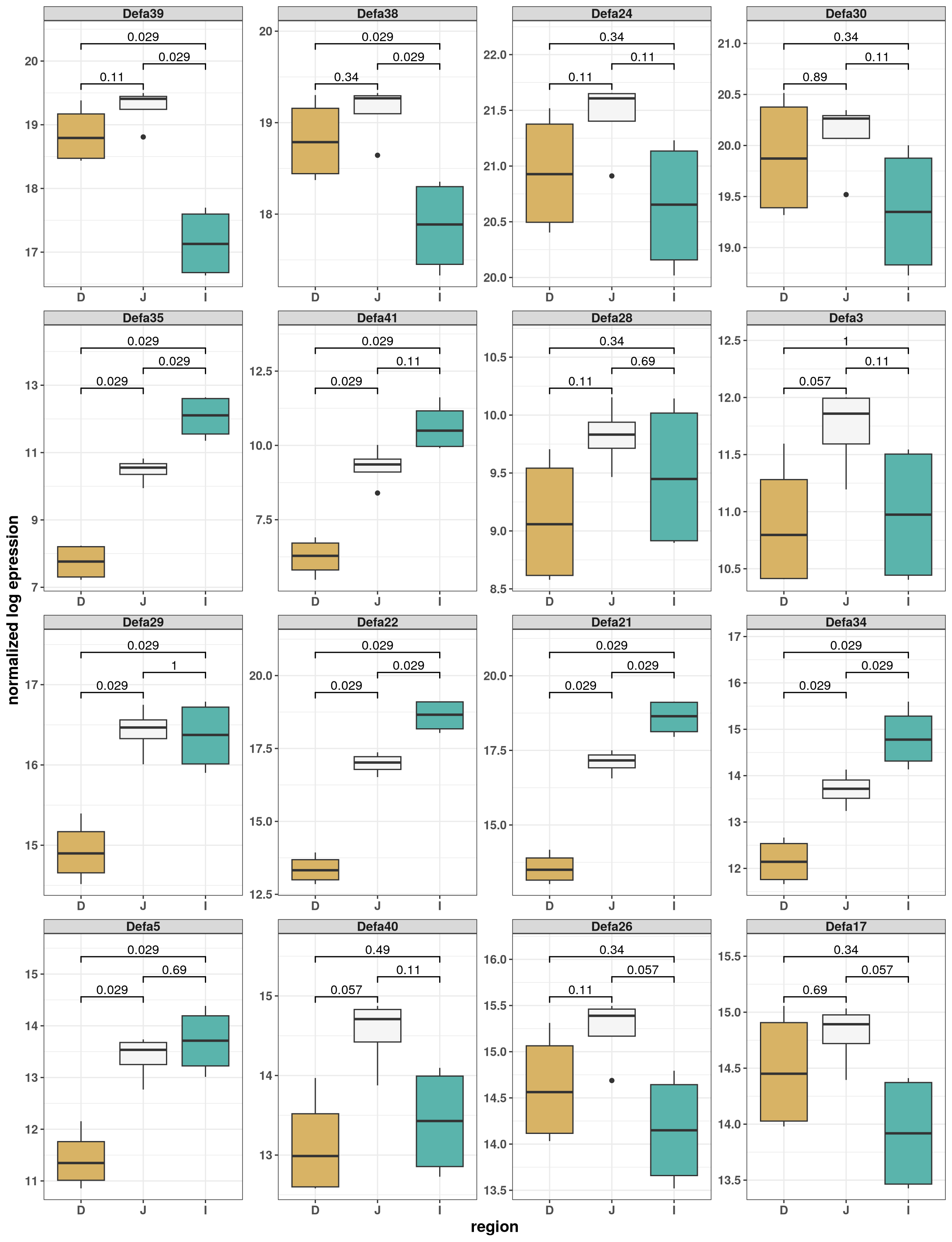

Supplement: Supplementary file 1 [file cells-13-01435-s001.zip › SupFigure_6.png]

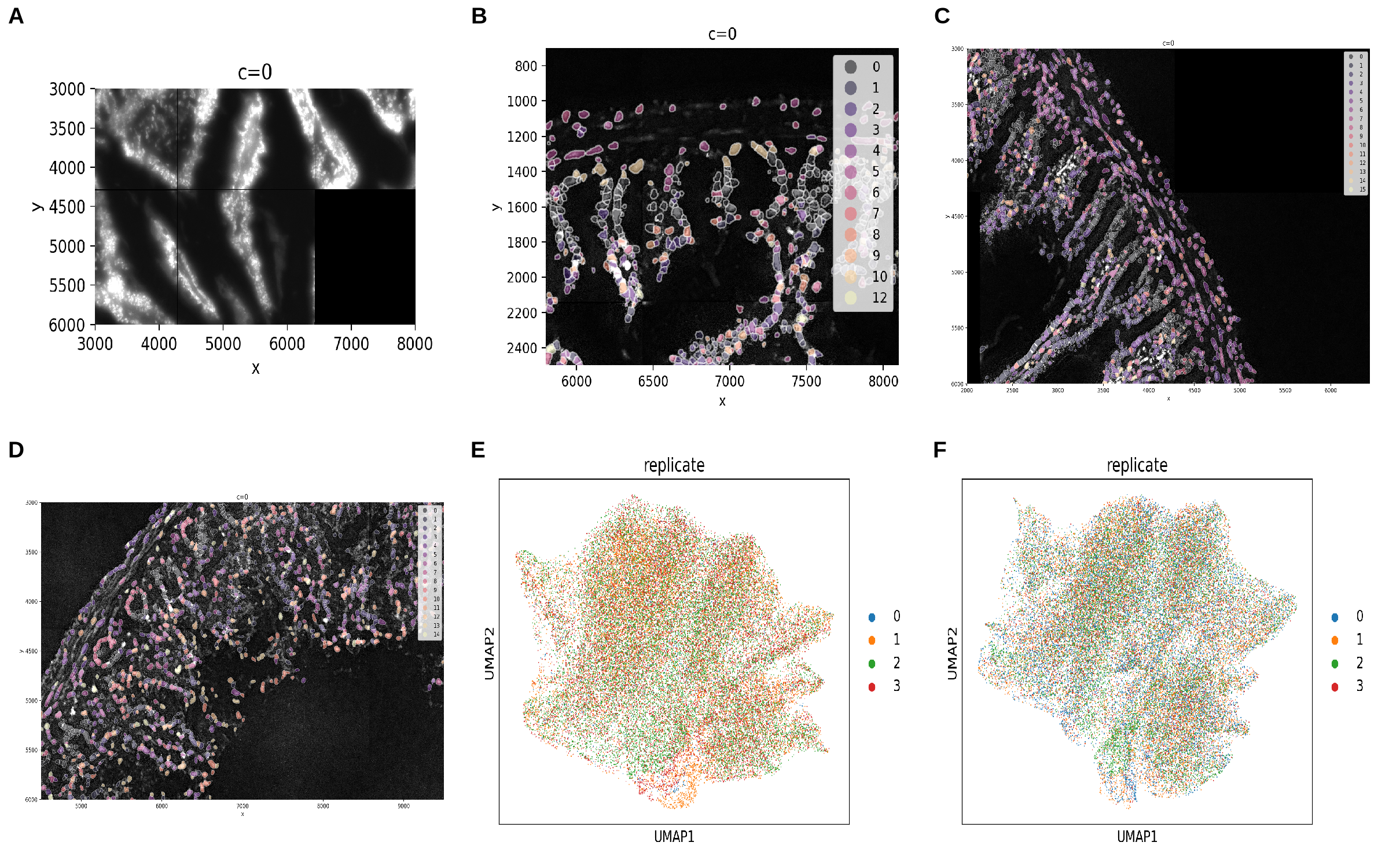

Supplement: Supplementary file 1 [file cells-13-01435-s001.zip › SupFigure_7.png]

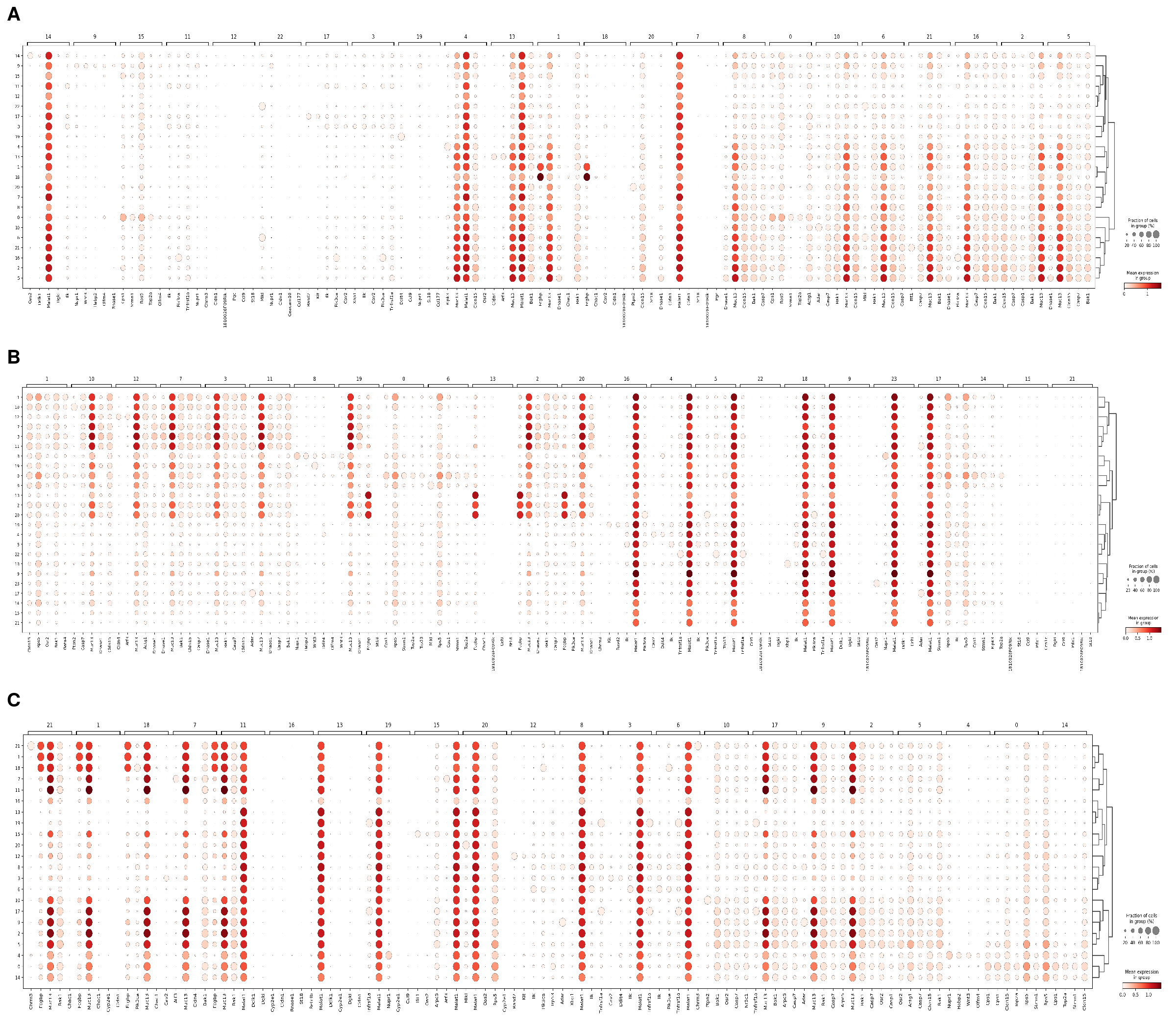

Supplement: Supplementary file 1 [file cells-13-01435-s001.zip › SupFigure_8.png]
